# Role of Immunological Cells in Hepatocellular Carcinoma Disease and Associated Pathways

**DOI:** 10.1021/acsptsci.3c00216

**Published:** 2023-11-08

**Authors:** Ram Aasarey, Kajal Yadav, Brijendra Kumar Kashyap, Sarit Prabha, Pramod Kumar, Anil Kumar, Janne Ruokolainen, Kavindra Kumar Kesari

**Affiliations:** † Department of Laboratory Medicine, All India Institute of Medical Science, New Delhi-11029, India; ‡ Department of Biotechnology, All India Institute of Medical Science, New Delhi-11029, India; § Department of Biotechnology Engineering, Institute of Engineering and Technology, Bundelkhand University, Jhansi-284128, Uttar Pradesh, India; ∥ Department of Biological Science and Engineering, Maulana Azad National Institute of Technology, Bhopal-462003, Madhya Pradesh,India; ⊥ Indian Council of Medical Research, National Institute of Cancer Prevention and Research (NICPR), l-7, Sector-39, Noida-201301, National Capital Region, India; # Department of Life Sciences, School of Natural Sciences, Central University of Jharkhand, Cheri-Manatu, Karmre, Kanke-835222, Ranchi, India; ∇ Department of Applied Physics, School of Science, Aalto University, FI-00076 Espoo, Finland; × Research and Development Cell, Lovely Professional University, Phagwara-144411, Punjab, India

**Keywords:** Cirrhosis, Hepatocellular carcinoma, HBV, HCV, NAFLD

## Abstract

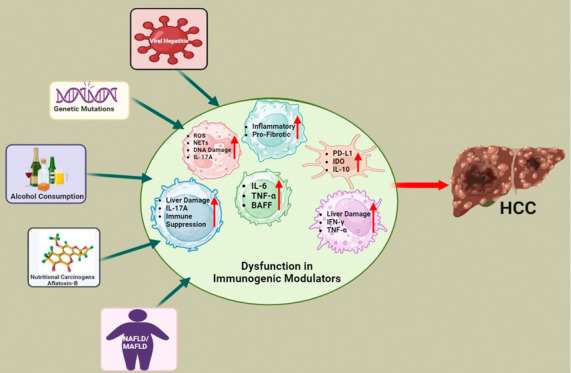

Hepatocellular carcinoma (HCC) remains one of the predominant causes of cancer-related mortality across the globe. It is attributed to obesity, excessive alcohol consumption, smoking, and infection by the hepatitis virus. Early diagnosis of HCC is essential, and local treatments such as surgical excision and percutaneous ablation are effective. Palliative systemic therapy, primarily with the tyrosine kinase inhibitor Sorafenib, is used in advanced cases. However, the prognosis for advanced HCC remains poor. This Review additionally describes the pathophysiological mechanisms of HCC, which include aberrant molecular signaling, genomic instability, persistent inflammation, and the paradoxical position of the immune system in promoting and suppressing HCC. The paper concludes by discussing the growing body of research on the relationship between mitochondria and HCC, suggesting that mitochondrial dysfunction may contribute to the progression of HCC. This Review focuses on immunological interactions between different mechanisms of HCC progression, including obesity, viral infection, and alcohol consumption.

Hepatocellular carcinoma (HCC) is a prominent cancer mortality etiology worldwide. A background of chronic liver disease and cirrhosis is present in 70% to 90% of individuals with HCC, with major risk factors including chronic infection with hepatitis B virus (HBV) or hepatitis C virus (HCV), alcoholic liver disease (ALD), and non-alcoholic steatohepatitis (NASH).^1,2^ Aflatoxin-contaminated food consumption, diabetes, obesity, certain hereditary conditions such as hemochromatosis, and some metabolic disorders are additional risk factors for developing HCC,^1,3^ as summarized in Figure 1. Due to the close correlation between hepatotropic viruses like HBV, HCV, and hepatitis D virus (HDV) and the development of HCC, its occurrence mimics the distribution of these viral infections around the globe.^4^ Additionally, these viral infections have an additive effect, increasing the risk of HCC by 2 to 6 times in the presence of HBV/HCV and HBV/HDV co-infections. Usage of alcohol also raises this danger further.^5,6^ Most of these factors can be avoided. Although a causal agent may frequently be identified, HCC remains an incredibly complex disorder with a dismal prognosis due to the numerous components involved in its etiology that all have a direct impact on patient features and disease progress.^7^ In addition, the geographic variation in etiology means that information from different countries is needed in order to optimize surveillance methods and develop effective chemoprevention strategies.^8^ With 800,000 death cases detected in 2012, liver cancer has the seventh-highest age-adjusted incidence rate 50 in the world. The prevalence of liver cancer worldwide continues to pose a significant challenge to global health, with a projected incidence rate exceeding 1 million cases in 2025. HCC accounts for approximately 90% of liver cancer cases^9^ and is responsible for about 800,000 deaths worldwide.^10^ It presents the fifth and seventh most significant cancer-related deaths in males and females, respectively. The rate of survival for HCC is very poor, below 20% globally.^10^ According to the U.S. National Cancer Institute’s SEER database, HCC ranks third globally and seventh in the U.S. in terms of cancer-related deaths, with a survival rate of about 5%.^11,12^

**Figure 1:**
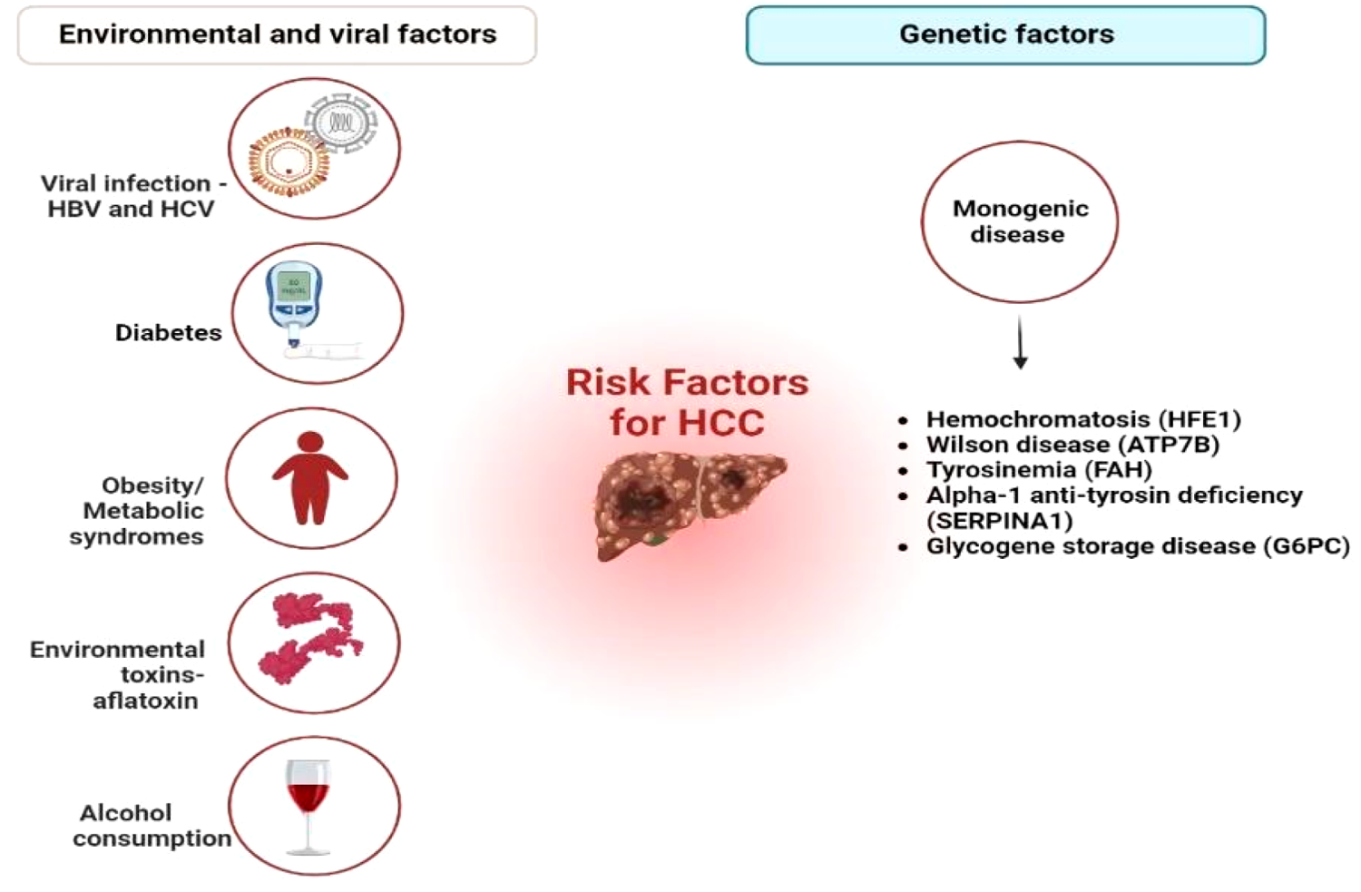
Major risk factors of hepatocellular carcinoma (HCC), including viral infections such as HBV and HCV, diabetes, obesity and other metabolic dysfunctions, alcohol consumption (30 g/day for males and 20 g/day for females), and environmental toxins such as aflatoxin-B1.

The malignant transformation and the progression of HCC disease are the results of a complex process known as HCC carcinogenesis, which can involve numerous modifications to a number of molecular pathways as well as genetic abnormalities.^13,14^ Here, we summarize the major risk factors, diagnostic approaches, treatment options, and emerging therapies along with the important immunological aspects and major molecular mechanisms involved in HCC carcinogenesis. The survival rate for HCC is very low. Studies cited in the references show that less than 20% of people diagnosed with HCC worldwide survive the disease.^10^

Local treatments such as surgical removal or percutaneous ablation effectively address early-stage HCC. However, the condition is typically detected when it has progressed to a more advanced stage. When faced with these situations, HCC is managed through systemic treatment, as shown in Figure 2, which represents the drug of choice for the treatment of HCC depending on the stage/condition. For HCC, immunotherapy drugs such as Nivolumab and Pembrolizumab, Atezolizumab and Bevacizumab, and Sintilimab and Camrelizumab have shown notable efficacy in both preclinical and clinical settings. However, there are also important drawbacks, such as response heterogeneity, resistance and relapse, and early-stage HCC. Future treatments for HCC patients may be more successful as a result of ongoing research that aims to better understand immunotherapy responses, combat resistance, and increase access to these medications. The therapy has been mainly centered on antivirals such as Sorafenib, a tyrosine kinase inhibitor (TKI), for the past decade.^15^ Chronic viral infections, especially hepatitis B and C, are significant risk factors for developing HCC. These viruses may result in long-term liver inflammation and damage, leading to liver disease progression, including cirrhosis and HCC. Antiviral therapies, such as the TKI Sorafenib, specifically inhibit viral replication, reduce liver inflammation, and slow the progression of liver disease. Antiviral drugs play a crucial role in HCC treatment and improve patients’ prognosis with chronic viral hepatitis by controlling the underlying viral infection.^16−18^

**Figure 2:**
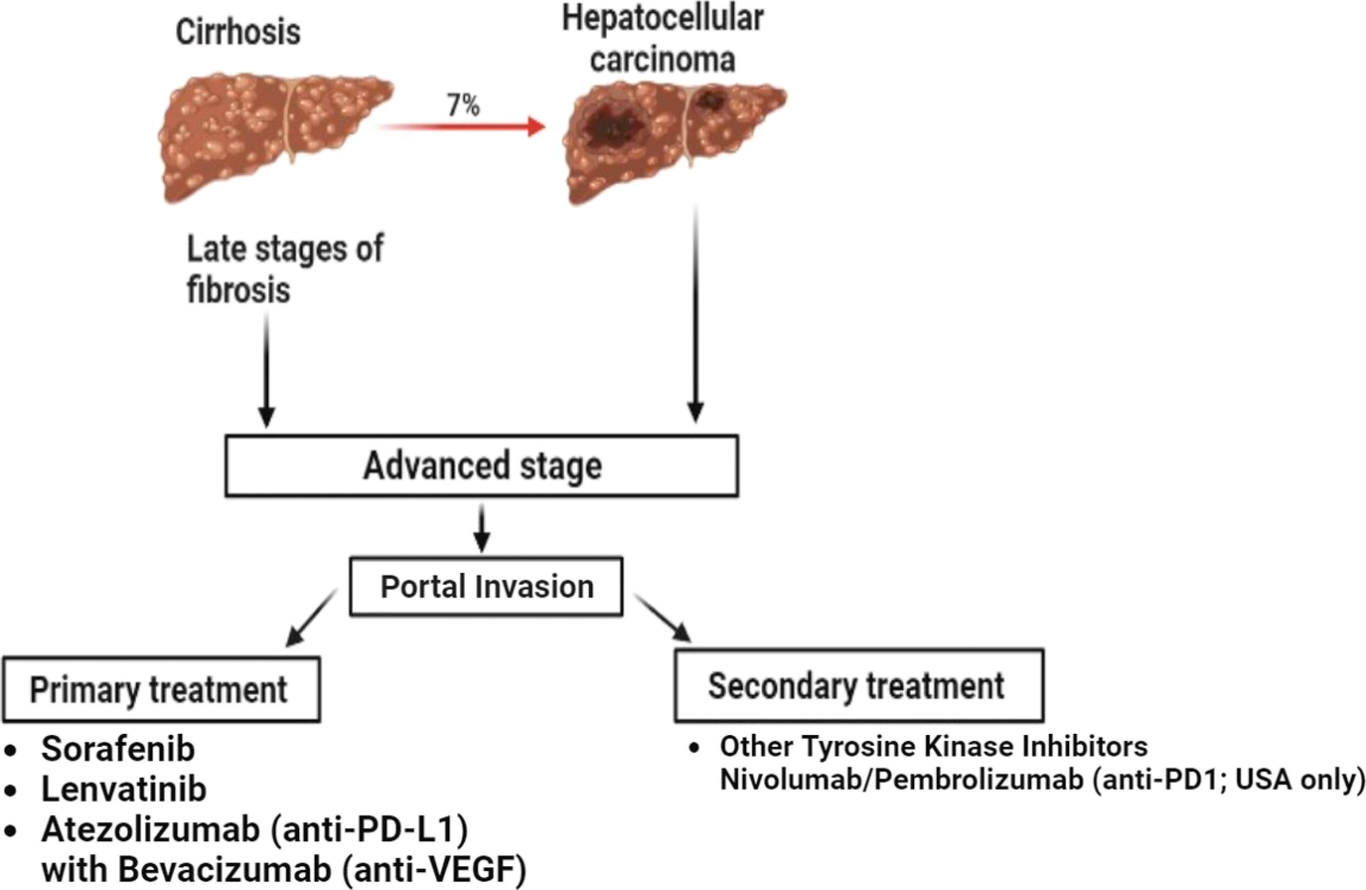
Drugs employed to treat advanced stages of HCC in primary and secondary care settings. Primary care medicines are the first-line therapy used when advanced HCC is diagnosed. Secondary care medicines are used when the first treatments are no longer effective or the disease develops following an initial response (PD-L1, programmed death ligand 1; VEGF, vascular endothelial growth factor).

Advanced HCC implies clinical challenges, but advancements in therapeutics help in management strategies.^19,20^ In current scenarios, clinical practices utilizing several TKIs, such as Lenvatinib,^21^ Cabozantinib,^22^ and Regorafenib,^23^ have been approved for first- and second-line therapies. These TKIs play an important role in the therapy landscape, acting as both first-line and second-line medicines. “First-line therapy” refers to the initial therapeutic technique used after a diagnosis of HCC. First-line medicines are designed to be the most effective and well-tolerated treatment options available, with a focus on slowing disease progression while maintaining the patient’s overall quality of life.^24^ On the other hand, “second-line therapy” is a treatment plan used when the first-line therapy fails to produce the expected results or when the disease worsens after an initial response. These therapies are intended to provide alternate ways, frequently with distinct mechanisms of action, to combat the disease when the first-line medication is no longer effective or well tolerated.^25^Table 1 highlights the critical function of TKIs such as Lenvatinib, Cabozantinib, and Regorafenib, which have been approved for both first- and second-line HCC treatments.

**Table 1:** Drugs Targeting Different Sites Approved for HCC

Drug Names	Trade Names	Developers	Targets and References	Therapeutic Line	Trial	Approval Date
**Sorafenib**(26,27)	Nexavar	Bayer and Onyx	PDGF, VEGFR, c-kit, Ras, ERK, MAPK	1	SHARP	16.11.2007
**Lenvatinib**(28,29)	Lenvima	Eisai Co.	VEGFA, VEGFC, KIT, RET	1	REFLECT	16.08.2018
**Atezolizumab** plus **Bevacizumab**(30)	Tecentriq and Avastin	Genentech Inc.	PD-L1 and VEGF	1	IMbrave150	29.05.2020
**Regorafenib**(31,32)	Stivarga	Bayer	STAT3, BRAF, and Tie2	2	RESORCE	27.04.2017
**Nivolumab**(33)	Opdivo	Bristol-Myers Squibb	PD-L1 and PD-L2	2	CheckMate-040	22.09.2017
**Cabozantinib**(34)	Cabometyx	Exelixis Inc.	VEGFR/MET Pahway	2	CELESTIAL	14.01.2019
**Pembrolizumab**(35)	Keytruda	Merck	PD1	2	KEYNOTE-224	09.11.2018
**Nivolumab** plus **Ipilimumab**(36)	Opdivo and Yervoy	Bristol-Myers Squibb	PD-1 + CTLA-4	2	CheckMate-040	10.03.2020

Despite recent evidence for targeted therapy and immunotherapy, prognosis of advanced HCC remains poor, with limited systemic therapy options and high recurrence rates after locoregional therapy.^37^ The pathophysiological mechanisms of HCC are not fully understood. However, HCC is thought to result from abnormal molecular signaling, genomic instability, and chronic inflammation.^38^

The role of the immune system in HCC is a current research focus around the globe, revealing a complex interplay where the immune system can have both beneficial and detrimental effects. Recent findings suggest that the immune system plays a dual contradicting role, contributing to improved survival in some cases while paradoxically promoting HCC progression in others.^39^ Over the past few decades, the literature concerning the connection between HCC and mitochondria has notably increased. According to these research findings, abnormal mitochondrial proteins in cancer cells may cause mitochondrial dysfunction, mitochondrial stress response, and mito-ribosome defects, which could lead to ROS production, metabolic reprogramming, and mitoformetic responses in the mitochondria of damaged liver cells.^40,41^ This Review discusses the immunological crosstalk between different mechanisms of HCC progression, like obesity, viral infection, and alcohol consumption.

## Risk Factors of HCC

### Hepatitis Virus

HBV and HCV belong to the hepadnavirus and flavivirus families, respectively. These viruses affect the liver and are transmitted through contaminated blood and bodily fluids, causing acute and chronic liver conditions characterized by necrosis.^42−45^ HBV infection in individuals possessing a competent immune system leads to self-limited transient hepatic manifestation. The findings reveal that the overwhelming majority, specifically over 95% of adults, can effectively eliminate disease and viruses.^46,47^ However, it is noteworthy that more than 90% of infants exposed to HBV at birth develop a permanent infection. The persistence of HBV infection is linked with diverse levels of chronic liver ailment, which frequently culminate in cirrhosis and HCC.^43^ Individuals who endure persistent infection with the HCV display ongoing late complications similar to cirrhosis or HCC.^48^

Acute hepatitis B typically results in full recuperation with a negligible or non-existent probability of HCC. Chronic hepatitis B infection poses a significant hazard, as it is recognized as the foremost predisposing factor for developing HCC due to several interconnected mechanisms, which include immune suppression, liver inflammation, production of oncogenic genes, fibrosis, and cirrhosis.^49−51^ It is complicated and multifaceted how persistent HBV infection affects the emergence of HCC. Understanding these pathways is essential for HCC prevention and creating focused treatments for those with persistent HBV infection. Chronic hepatitis is distinguished by enduring hepatic illness accompanied by hepatocyte regeneration involving cellular DNA synthesis and inflammation that encourages mutagen production, unintentionally causing genetic and chromosomal aberrations that can potentially instigate the onset of HCC. Numerous studies have reported that the initial stage of HBV infection involves the liberation of HBV envelope polypeptides alongside several cellular membrane proteins—PreS1,^52^ endonexin II,^53^ interleukin-6 (IL-6),^54^ annexin V,^55^ apolipoprotein H,^56^ the transferrin receptor,^57^ and gp180/carboxypeptidase D for duck hepatitis B virus (DHBV).^58,59^ It has been discovered that the presence of heparan sulfate proteoglycans on the surface of cells aids in the initial binding of the HBV to hepatocytes through low-affinity binding involving the S protein antigenic loop, which subsequently facilitates the process of invasion.^60^ The HBV has been found to recognize the sodium taurocholate co-transport polypeptide (NTCP), which is also known as SLC10A1, as a receptor.^61^ The internalization of HBV is observed to occur through the caveolae-mediated endocytic pathway, primarily under neutral pH conditions. It circumvented the acidic endosomal compartment of the clathrin-mediated pathway.^62^ Upon their release, nuclear particles are subsequently transported to their intended destination, the cell nucleus. The influx of core particles into the nucleus is facilitated by the nuclear pore complex (NPC), which enables the transportation of particles with a diameter of up to 39 nm.^63^ The interaction between core particles and NPCs necessitates the phosphorylation of the core protein, which in turn results in the exposure of the nuclear localizing signal (NLS) located in the C-terminal domain (CTD).^64^ The externally displayed NLS can bind with the importin α/β transport receptor. This interaction is facilitated by introducing the core particle into the nuclear basket,^63^ which can subsequently bind to nucleoporin 153, an integral constituent of the NPC.^65^ At this juncture, the degradation of nuclear particles results in the liberation of genomic DNA into the nucleoplasm.

In this context, the relaxed circular viral DNA genome is liberated within the nucleus. Cellular polymerases facilitate the repair of viral DNA, forming covalently closed circular (CCC) DNA. The latter serves as episomal mini-chromosomes that constitute the transcription template for viral replication. The CCC DNA molecule also serves as a genetic blueprint for synthesizing four capped polyadenylated RNAs, which are responsible for producing viral proteins, both structural and non-structural. One of the consequential transcripts of the HBV is characterized by a length of 3.5 kilobases, surpassing the size of the viral genome itself, and is consequently responsible for synthesizing critical proteins, namely the viral core and polymerase. In the cytoplasm, the current transcript functions as a genomic RNA polymerase that is encased in the core protein. The viral replication process entails the interiorization of pre-genomic RNA within the capsids, after which the RNA undergoes reverse transcription, generating single-stranded DNA copies. These DNA copies subsequently act as templates for synthesizing a secondary strand. The process of DNA synthesis facilitates the production of circular and partially double-stranded (ds) DNA genomes. Viral capsids harboring dsDNA may undergo two distinct pathways, whereby they either regress to the nucleus to expand the viral CCC DNA genome or translocate to the endoplasmic reticulum where they associate with viral envelope proteins, egress into the lumen, and penetrate host cells as infectious virions.^46,66^ HBV DNA integration causes HCC primarily through three mechanisms: (1) chromosome instability of the HBV-integrated DNA; (2) modification of proto-oncogene expression or function to facilitate the growth of liver cancer; (3) expression of a mutant HBV protein that has been incorporated.^51,67^

HCV is a viral pathogen that spreads through the bloodstream and gains access to the liver. A previous study investigated the binding mechanism of HCV E1.E2 glycoproteins with various host cellular receptors. It first attaches to hepatocytes with the help of LDL-R and HSPG receptors (low-affinity binding), then it binds with affinity to human scavenger receptor class B type I (SR-B1),^68,69^ which mediates binding to CD81 receptors.^70,71^ The CD81 molecule present on the cellular membrane of the host acts as a receptor for the viral E2 glycoprotein, allowing for viral particle binding and subsequent entry into hepatocytes.^72^ The HCV also binds to other receptors, including dendritic cell (DC)-specific intercellular adhesion molecule 3-grabbing non-integrin (DC-SIGN), the related liver- and lymph node-specific L-SIGN, and claudin-I (CLDNI). These receptor interactions are significant, as they facilitate viral attachment and entry into host cells, playing a crucial role in the process of HCV infection.^73,74^ The binding activity is organ-specific and confined to the liver and lymph nodes. Furthermore, existing literature has demonstrated the ability of CLDN6 and CLDN9 to substitute CLDN1 as entry factors for HCV into cells other than hepatocytes in the human body.^75^ The presence of two hypervariable regions, namely HVR-1 and HVR-2, has been observed in E2 glycoprotein, where HVR1 is found primarily in the amino-terminal portion whereas HVR2 is found in the C-terminal portion.^76,77^ These regions undergo frequent mutations, which have been attributed to antigenic alterations of the virus that contribute to immune evasion against viral antibodies and cytotoxic T lymphocytes (CTLs). HCV exhibits a considerable mutation rate, which arises from the deficient proofreading capability of its RNA-dependent RNA polymerase (RDRP). Consequently, HCV manifests in various closely affiliated viral strains within the affected hosts. During viral persistence or in chronic infections, RDRP also contributes to seroconversions to evade the host immune response. The taxa are recognized as subtypes of the HCV. The non-structural (NS) proteins present in HCV, namely NS3 and NS4A, establish an intricate complex that triggers the activation of the NS protease domain while guiding the cleavage process of IPS-1. Following the cleavage process, IPS-1 is rendered incapable of downstream signaling, preventing the activation of IRF-3 and NFκB. This leads to a lack of production of IFN-β and the expression of ISG in infected cells.^78^ HCV has developed numerous tactics to mitigate the natural killer (NK) cell reactions of the host. Remarkably, activated NK cells have been shown to facilitate hepatic injury, while non-activated or impaired NK cells fail to impede virus invasion.^79^ The significance of the NS5A and E2 regions bears considerable weight in the context under consideration. The NS5A protein plays a role in facilitating viral replication, impairing the function of PKR,^80^ hindering apoptotic pathways, binding to growth factor receptor binding protein 2,^81^ and inducing anti-inflammatory IL secretion.^82^ Similarly, the E2 protein functions to inhibit PKR activity.^83^ The interaction between HCV E2 proteins and DCs leads to DC maturation. Various hepatitis C viral proteins, namely core, NS3, NS5A, and NS5B proteins, have been demonstrated to impede the functionality of DCs.^84^ The empirical investigation has demonstrated that, in patients with chronic HCV infections, there exists an inhibition of DCs^85^ and a decrease in the function of CD4^+^ and CD8^+^ T cells.^86^ The E2 protein has been found to elicit analogous impacts in diverse cell populations, including T cells, B cells,^87^ hepatocytes,^88^ and hepatic stellate cells (HSCs),^89^ as evidenced in previous studies. The etiology of HCV infection is intricate and subject to various metabolic activities that profoundly impact liver function, induce inflammation, and impede the host’s immunological response. HCV infects and replicates within hepatocytes. Over time, inflammation and liver cell damage from this ongoing viral replication may occur.^90^ HCV has found strategies to get around the immune system of the host. Infection that persists and chronic liver inflammation are both caused by this immune evasion.^91^ Reactive oxygen species (ROS), which are toxic, can produce oxidative stress within liver cells because of HCV infection. The liver scarring conditions cirrhosis and liver fibrosis, both of which are advanced stages, are mostly brought on by chronic HCV infection.^92^ These variables help liver disease proceed, making HCV a significant risk factor for diseases including cirrhosis and HCC. The pathogenic effects of HCV involve the participation of both innate and adaptive immune systems, with particular emphasis on the role of cytotoxic lymphocytes in determining the clearance or longevity of viral particles. Moreover, the persistence of diverse HCV factors, including viral proteins, strain genotype, and hepatic metabolism, modulates infection.

The HBx regulatory proteins have demonstrated the capability of disrupting the standard control of cell cycle progression by their capacity to engage in binding interactions with numerous cellular partners and initiate transcriptional and signaling cascades.^93^ The integration of the HBV frequently impacts HBx, which is frequently eliminated toward its 3′ terminus, thereby resulting in the manifestation of HBV proteins truncated at their C-terminus. These truncated proteins have been demonstrated to augment the invasiveness and metastatic potential of HCC cells. The application of HBx has been found to have beneficial effects in combating age progression. This process curtails the propagation of impaired cells while decreasing the likelihood of malignancy by activating tumor suppressor genes. Several mechanisms underlying this phenomenon have been elucidated in the literature.^94^

One such mechanism is the inhibition of the nucleotide excision repair and transcription-coupled repair functions of the P53 protein. The observed sample showed a reduction in the levels of ASPP1 and ASPP2, which are proteins known to activate P53. The deactivation of the tumor suppressor RB occurs through the repression of CDK inhibitors INK4A and P21 via the process of promoter methylation.^50^ HCV has been postulated to potentially promote carcinogenesis due to the propensity for chronic inflammatory reactions and/or the activation of HSCs that trigger fibrosis within the liver. Chronic hepatitis is correlated with an alteration in the signaling of tumor growth factor-beta, leading to a transition from tumor suppression to the development of fibrosis and carcinogenesis.^95^ The proliferative alterations within hepatic tissues, as a consequence of prolonged inflammation resulting from hepatitis, are intrinsically linked to recurrent cellular demise and subsequent rebirth.^96^ The repetitive progression of cell cycles is closely linked with the accrual of genetic alterations, which can foster the malignant transformation of hepatocytes through a complex, multi-phase pathway. Individuals who have HCV genotype 3 are found to have a heightened susceptibility to HCC. This association implies that genotype 3 of HCV may have a significant role in a person developing HCC, independent of other factors. The induction of HSC proliferation and resulting fibrosis has been observed in vitro in cell culture systems upon stimulation with serum obtained from patients afflicted with HCV.^97^ The HCV core protein potentially possesses the ability to facilitate the development of cancer. Chronic hepatocellular injury is a pathological state that may lead to cancer development by facilitating cellular mechanisms, such as HBV and HCV, that impede cellular DNA synthesis, augment the production of inflammatory mutagens, and impair essential cellular processes such as detoxification and repair. The prolonged occurrence of these events has the potential to give rise to numerous genetic and chromosomal modifications in the process of HCC development.

### Alcohol Consumption

The International Agency for Research on Cancer (IARC) has identified alcohol and alcohol-associated aldehydes as type 1 carcinogens. Alcohol consumption has been identified as a causative agent of HCC in human beings, akin to other organ malignancies including colorectal, female breast, pharynx, larynx, oral cavity, and other cancers.^98,99^ This assertion posits the fact that alcohol consumption is irrefutably associated with the development of HCC, a form of cancer that affects the liver.^100^ According to the World Health Organization (WHO), an estimated 4.1% of individuals aged 15 and above, equivalent to around 280 million people, meet the criteria for alcohol use disorders, encompassing both alcohol dependence and harmful alcohol consumption.^101^ The frequency of occurrence is comparable to that of hepatitis B, and it is 4-fold compared to that of hepatitis C.^102^

Similar to hepatitis C and hepatitis B, alcoholic cirrhosis confers significant susceptibility to the development of HCC. According to existing literature, a percentage range of 10–20% has been reported pertaining to the likelihood of cirrhosis development among individuals with a history of heavy alcohol consumption.^103^ The consumption of alcohol at a rate of 30–50 g/day has been found to escalate the likelihood of developing cirrhosis, while a corresponding rate of 60–100 g/day has been correlated with an increased risk of HCC.^99,104−106^ According to existing literature, consistent consumption of 60–80 g/day of alcohol in men and 20 g/day in women over a decade has been linked to an elevated likelihood of developing liver cirrhosis.^107^

Upon ingestion, the chemical compound ethanol is readily absorbed within the small intestine and subsequently undergoes metabolic processes within the liver.^108^ The hepatic cytoplasm undertakes the process of alcohol dehydrogenase (ADH)-mediated ethanol metabolism, which results in the generation of acetaldehyde as the primary product. Subsequently, acetaldehyde undergoes mitochondrial entry and is oxidized to acetate by the action of aldehyde dehydrogenase (ALDH) localized within these organelles. The main pathway of alcohol metabolism involves the process of NAD hydrogenation, leading to the accumulation of NADH. Alcoholic steatosis is attributed, to a certain extent, to excessive elevation of the NADH/NAD^+^ ratio. The process of ethanol metabolism is facilitated by the involvement of various cellular components, including the endoplasmic reticulum and peroxisomal catalase. A pathway reliant upon CYP2E1 enables the catalysis of ethanol to acetaldehyde, concomitant with the generation of ROS. These ROS comprise hydroxyethyl groups, superoxide anions, and hydroxyl radicals.^109^ Aldehyde dehydrogenase 2 (ALDH2) is responsible for metabolizing acetaldehyde, a toxic byproduct of alcohol metabolism. When ALDH2 is depleted, acetaldehyde accumulates in the liver and activates multiple oncogenic pathways (JNK, STAT3, BCL-2, and TAZ), promoting HCC.^110^ Increased oxidative stress due to acetaldehyde accumulation can disrupt cellular processes and lead to lipid peroxidation. This can lead to the accumulation of lipids, mainly triglycerides, in liver cells. Fat accumulation in liver cells is a characteristic feature of fatty liver disease. The inflammation, steatohepatitis, is also often associated with fatty liver disease.^111^ Transaminases such as alanine transaminase (ALT) and aspartate transaminase (AST) are enzymes usually present in liver cells. When liver cells are damaged, they release these enzymes into the blood, leading to increased serum transaminase levels.^112^ The impaired functionality of ALDH2 leads to a mitigated form of hepatic steatosis and a reduction in serum transaminase levels. However, it unexpectedly exacerbates the inflammatory response and fibrosis in the liver.^113^

Chronic alcohol ingestion precipitates the oversaturation of enzymatic pathways, provoking anomalous accumulation of acetaldehyde, culminating in cytotoxic sequelae. The pivotal process underlying the progression of alcohol-related liver cancer in oxidative stress, which is triggered by the elevated levels of iron accumulation resulting from alcohol metabolism, inflammation, and ROS. The ROS instigates harm to the cellular macromolecules and participates in the advancement of liver cancer by generating lipid peroxides, among which 4-hydroxy-nonenal prevails. The accrual of ROS leads to consequential alterations in DNA’s structural and functional attributes, consequently influencing gene functions and processes such as replication and transcription. The implications of ROS-induced modifications in DNA are particularly pertinent in relation to the progression and accentuation of cancer.^114,115^

The disturbance of the gut microbial composition, metabolome, and gut epithelial barrier caused by chronic and “binge” alcohol consumption has been demonstrated to have a degrading effect on nutrient absorption in the human body.^116^ The human gut microbiome refers to a complex community of bacteria, viruses, fungi, and archaea that fluctuates depending on host genetics less so than depending on environmental conditions (such as nutrition and medicines).^117,118^ Through its detrimental impact on gut integrity, alcohol-induced dysbiosis contributes to the emergence of both acute (such as alcoholic hepatitis) and chronic (such as alcohol-related cirrhosis) liver disorders. These diseases are brought on by disturbances of the intestinal mucous barrier, which is crucial for the gut’s immune system. Claudins, occludin, and zona occludens, which form apical “tight junctions” between adjacent enterocytes in this barrier, prevent unwanted translocation of luminal contents into the portal circulation, including pathogen-associated molecular particles and bacterial endotoxins.^119^ These tight connections have been shown to be damaged in alcohol-induced dysbiosis. As a result, the gut barrier is further compromised by the ensuing immunological dysfunction and rise in circulating pro-inflammatory cytokines including tumor necrosis factor (TNF) and IL-1.^120^ In this way, the ingestion of alcohol has been shown to augment intestinal permeability, facilitate the relocation of lipopolysaccharide (LPS) and peptidoglycan from gut bacteria to the hepatic system, and induce an inflammatory response.^121^

Alcohol-related dysbiosis invariably has an impact on the gut metabolome and causes significant changes in short-chain fatty acids (SCFAs), which are crucial for maintaining the integrity of tight junctions. SCFAs, which are fatty acids with less than six carbon atoms, result from the gut microbiota’s anaerobic fermentation of food fibers that are not digested.^122^ The feces metabolome of people with alcohol use disorders showed a decrease in SCFAs, probably partly due to dysbiosis, which affects SCFA-producing bacteria like Faecalibacterium.^123,124^ Both non-essential amino acids like glutamic acid and necessary, dietary-obtained amino acids like lysine are altered. This is thought to be caused by dysbiosis, which disrupts the co-metabolism of the host and microbe.^125^ This metabolic imbalance may play a role in the generation of increased levels of ROS and toxic intermediates and alter gut permeability.^126^ Within Kupffer cells (KCs), LPS engages with Toll-like receptor (TLR) 4, instigating the synthesis of key pro-inflammatory cytokines, including IL-6 and TNF-α. The molecules are crucial in facilitating the mediation of various signaling pathways. The subject was involved in the progression of HCC.^93,94^ NF-κB, a crucial regulator of gene transcription in inflammatory processes, is activated during the development of ALD and controls the expression of a variety of pro-inflammatory cytokines and receptors, including TNF-α, IL-1, IL-6, EGF, and TLRs.

The latter phenomenon plays a crucial role in the development of cancer, as it enhances the buildup of ROS and triggers the activation of STAT3. An association between the allelic variant IL-6-174G and HCC has been observed among patients with ALD. Moreover, the proliferative and invasive characteristics of neoplastic cells are augmented by EGF. Moreover, the 50 untranslated regions of EGF gene expression give rise to functional polymorphisms that result in the substitution of A to G in the late region. The present study has established a significant correlation between the presence of HCC in Caucasian populations with alcoholic or HCV-associated liver disease and the G allele, which is known to lead to increased levels of transcription.^127−129^ Alcohol abuse is typified by the hepatocellular accumulation of lipids, predominantly comprised of triglycerides, phospholipids, and cholesterol esters. Numerous single-nucleotide polymorphisms, which were first identified through genome-wide association studies, may potentially play a role in the development of HCC. Alcohol-related metabolites have been demonstrated to exert oxidative stress and direct mutagenic effects as well as abnormal levels of DNA and protein methylation on hepatocytes. These findings implicate the immune system in the advancement and development of HCC.

### Aflatoxin-B1

The mycotoxin known as aflatoxin B1 (AFB1) is synthesized by the ubiquitous fungi *Aspergillus flavus* and *Aspergillus parasiticus*. Mycotoxins are observed to be present in various food items, including rice, corn, oilseeds, dried fruits, and peanuts. Such mycotoxins tend to accumulate in food materials that are exposed to hot and humid environments along with unsanitary storage conditions.^130^ Furthermore, it has been posited that substantial consumption of AFB1 among patients afflicted with HBV represents an auxiliary perilous element that advances the likelihood of HCC emergence.^3,131^

AFB1 consumption and HBV infection act synergistically to induce DNA damage and mutations and disrupt DNA repair by producing an unstable reactive intermediate that can bind to DNA, forming DNA adducts. These adducts can cause mutations in critical genes, including tumor suppressor genes and oncogenes.^132^ AFB1 and HBV infection create a pro-inflammatory environment and reduce the immune system’s ability to fight cancer cells.^133,134^ These molecular mechanisms combine to increase the risk of developing HCC in individuals with both risk factors. Regions exhibiting a marked prevalence of HCC and elevated consumption of aflatoxin are typically co-localized with regions where there is an endemic prevalence of HBV infection. It has been observed that individuals who have been exposed to both HBV and AFB1 are at the highest risk for developing HCC.^135^ Genetic anomalies in human cancers have been noted, specifically the prevalence of somatic mutations in the p53 tumor suppressor gene. Numerous investigations have supported the high incidence of p53 mutation in HCC. El Far and colleagues undertook a study to investigate the relationship between p53 mutations and a range of predictive factors encompassing tumor grade, α-fetoprotein, and liver function tests in cases of HCC originating from Egypt. The aim was to provide insight into the potential impact of these factors on the bottom line of HCC pathogenesis.^136^ The authors observed that the identification of p53 led to a rise in the rate of HCC prediction from 79.5% to 86.3%. Moreover, a notable affirmative association was observed between p53 mutations and tumor dimension in neoplasm grades II and III. It can be surmised that measuring serum levels of P53 protein may hold potential as a reliable and non-invasive means of screening for susceptibility to HCC.^137^

AFB1 can trigger HCC by eliciting particular mutations in codon 249 of the p53 gene, which encodes the tumor suppressor protein P53.^138^ However, it is noteworthy that this mutation has also been detected in patients who possess a prior history of exposure to HBV.^139^

### Obesity

Obesity has been acknowledged as a noteworthy contributing determinant in various oncological ailments,^140^ notably HCC, and to a certain extent in malignancies related to being overweight. The presence of aberrant gut microbiota has been linked to the occurrence of obesity, resulting in an augmentation of bacterial lipoteichoic acid (LTA) levels. Obesity-related metabolic dysfunction, chronic inflammation, and increased energy extraction from diet can all be caused by abnormal gut microbiota.^141^ Due to this dysbiosis, Gram-positive bacteria may overproduce LTA, which can stimulate the immune system and worsen the inflammatory response by triggering the release of inflammatory cytokines such as TNF-α and IL-6.^142^ This immune activation can further contribute to chronic inflammation and insulin resistance (IR), which are hallmark features of obesity-related metabolic dysfunction.^143^

LTA has the potential to facilitate the development of HCC by augmenting the senescent HSCs.^144^ Furthermore, LTA collaborates with deoxycholic acid (DCA), a secondary bile acid that is generated by the microbiota in the gastrointestinal tract, to govern the expression of SASP factor and cyclooxygenase-2 (COX2) via the participation of TLR2.^145^ The onset of obesity resulting from exposure to a high-fat diet (HFD) has been found to interfere with the functioning of the cytotoxic CD8^+^ T cells in the tumor microenvironment. This interference is caused by the alteration of fat uptake into tumor cells that is initiated by reduced expression of prolyl hydroxylase-3 (PHD-3).^146^ Obesity has been observed to potentially exert a regulatory effect on glucose metabolism and facilitate the progression of HCC. Saturated fatty acids, for instance palmitic acid, have a discernible impact on cancer stem cell attributes, ROS creation, and glucose metabolism, therefore advancing HCC inception and advancement.^147^

The condition of obesity has been consistently linked with the onset and development of metabolic syndromes, including IR and type 2 diabetes mellitus (T2DM). Additionally, it has also been found to be associated with different types of non-neoplastic liver diseases, such as non-alcoholic fatty liver disease (NAFLD), NASH, liver fibrosis, and cirrhosis.^148^

NAFLD is commonly linked with conditions such as T2DM and dyslipidemia.^149,150^ Excessive caloric consumption, genetic predisposition, or co-morbidities causing fat accumulation can result in hepatic dysfunction, as the liver displays a heightened synthesis of triglycerides without their subsequent excretion. The accumulation of triglycerides within hepatocytes, resulting in the development of hepatic steatosis, is a common manifestation of a fatty liver.

The prolonged inflammatory mechanisms inherent in NAFLD and NASH constitute the fundamental underpinnings for the emergence and progression of HCC.^151^ Furthermore, it has been noted that the pathogenesis of HCC attributed to NAFLD involvement of both innate and adaptive immune cells. The immune mechanisms underlying the development of HCC in NAFLD remain largely undetermined. Various studies have established the crucial participation of hepatic macrophages, comprising resident KCs and migrant monocyte-derived macrophages, in the development of NASH.^152^ The activation of KCs is a crucial component of tumor development during the preliminary phases of carcinogenesis induced by chemicals.^153^ Upon the formation of primary tumors, it has been found that there exists a significant inflow of tumor-associated macrophages, which are involved in the promotion of HCC.^154^

Numerous reports have indicated the heightened involvement of adaptive immunity, particularly T and B lymphocytes, in the progression of NAFLD-associated HCC. This is demonstrated through the amplified recruitment of adaptive immune cells to the liver in affected patients. As an illustration, the utilization of whole-exome sequencing for the liver of a patient with HCC related to NAFLD had established the presence of gene signatures that are linked with T lymphocytes, thus indicating a heightened accumulation of hepatic T cells. Several studies have demonstrated that the HCC case associated with NAFLD and cirrhosis displayed immune depletion which led to a dysfunctional state, possibly due to stimulation by tumor antigens.^155^ Another study presents a mouse model of HCC that is associated with NAFLD and exhibits metabolic disorders. Our findings suggest that intrahepatic CD8 T cells can be activated and express CD44 and CD69 in this model. Additionally, these T cells may induce liver injury through direct interaction with hepatocytes. The mouse model in question reveals that genetic ablation of CD8 T cells engenders the emergence and advancement of liver injury, NASH, and HCC, leading to the conclusion that CD8 T cells play a direct role in the progression of disease.^156^ NASH programming incites CD8 T cells to assume an exhausted, activated phenotype and exhibit elevated expression levels of programmed cell death protein-1 (PD-1) upon exposure to metabolic stimuli such as IL-15. It induces non-specific hepatic cell death and progression.^157^

The effect of CD8^+^ T and NKT cell depletion to protect from HCC development may be attributed to the inability to attain the NASH phase. In contrast, the CD8^+^ T cells provide protection in HFD-fed MUP-uPA mice that lack immunoglobulin A (IgA) against HCC induced by NASH. In this study, it was observed that CD8^+^ T cells in mice demonstrated a restricted impact on NASH progression. Conversely, resistance to HCC was linked to a decrease in the quantity of exhausted CD8^+^ T cells. Hence, the inhibition of PD-L1 leads to the restoration of the depleted T cells in MUP-uPA mice subjected to HFD, which generates an elevation in anti-tumor immune response and a decline in tumor occurrence at a level of 92. Consequently, it can be posited that CD8^+^ T cells perform a crucial function in suppressing tumor growth in the HFD-fed MUP-uPA murine model. Additionally, in alternative models of HCC induced by NASH,^158^ Cd8a-deficient mice developed a higher tumor burden.

In contrast to the observed accumulation of CD8 T cells, it has been observed that dysregulation of lipids is associated with a decline in CD4 T cells in the hepatic tissues of mice affected by NASH, both in the presence and in the absence of tumor development. This condition is often attributed to the use of methionine- and choline-deficient l-amino acid diets. Research has provided empirical evidence indicating that it can induce an acid-deficient diet. The induction of CD4^+^ T cell death through mitochondrial ROS production by fatty acids has been observed, with subsequent research indicating that ROS knockout serves to limit CD4^+^ T cell loss and minimize tumor burden.^159^ It has been hypothesized that the impact of CD4^+^ T cells on tumor growth is primarily attributed to their capacity to initiate immune responses that are specific to the tumor. Furthermore, an opposing impact of CD4^+^ T cells has been documented in an alternative model of HCC induced by NASH. Undoubtedly, the facilitation of NASH development and advancement to HCC is attributed to TH17 cells that produce IL-17A through IL-17A-induced signaling in myeloid cells.^160^

Recent research has indicated that, aside from T cells, B cells that secrete IgA also make a significant contribution to the advancement of HCC associated with NAFLD. A correlation between tumor progression and the quantity of B cells that infiltrate tumors is observed in human subjects.^161^ Based on our analysis, HCC patients displaying minimal infiltration of plasma cells within their tumors exhibit a positive prognostic outcome.^162^ The administration of HFD to transgenic mice expressing the urokinase-type urinary plasminogen activator protein, which is regarded as a valid model for NAFLD-associated HCC, elicits a significant upregulation of the production of IgA-secreting cells, as well as cytotoxic CD8 T cells that mediate an immune response to protect against tumor-related damage. B cells positive for IgA expression exhibit the expression of PD-L1 and stimulate the production of the immunosuppressive cytokine IL-10, consequently inhibiting the functioning of CD8 T cells.^158^ A particular category of B cells, known as regulatory B cells, has the ability to impede the immune response against tumors. This is accomplished through the production of IL-10 and by facilitating the proliferation of HCC cells via direct engagement with the malignant cells.^163^

The impact of innate immune cells on the development of HCC in NASH may also be substantial through mechanisms involving sterile cell death processes resulting from lipotoxicity in hepatocytes as well as causing altered gut–liver axis function, chronic inflammation, oxidative stress, and immune dysregulation and encouraging hepatocyte survival and proliferation.^164,165^

Neutrophils have been shown to facilitate the progression of NASH by releasing neutrophil extracellular traps (NETs). Limiting the production of nuclear envelope-derived vesicles has been found to effectively mitigate the inflammation associated with NASH and decrease the likelihood of HCC development that may arise due to NASH.^166^ This may be attributed to the constrained incidence of NASH. While the potential influence of other myeloid cell populations, including KCs, in the context of NASH-induced HCC remains unexplored, the existing scholarship concerning macrophage involvement in hepatocellular carcinogenesis is vast.

**Figure 3:**
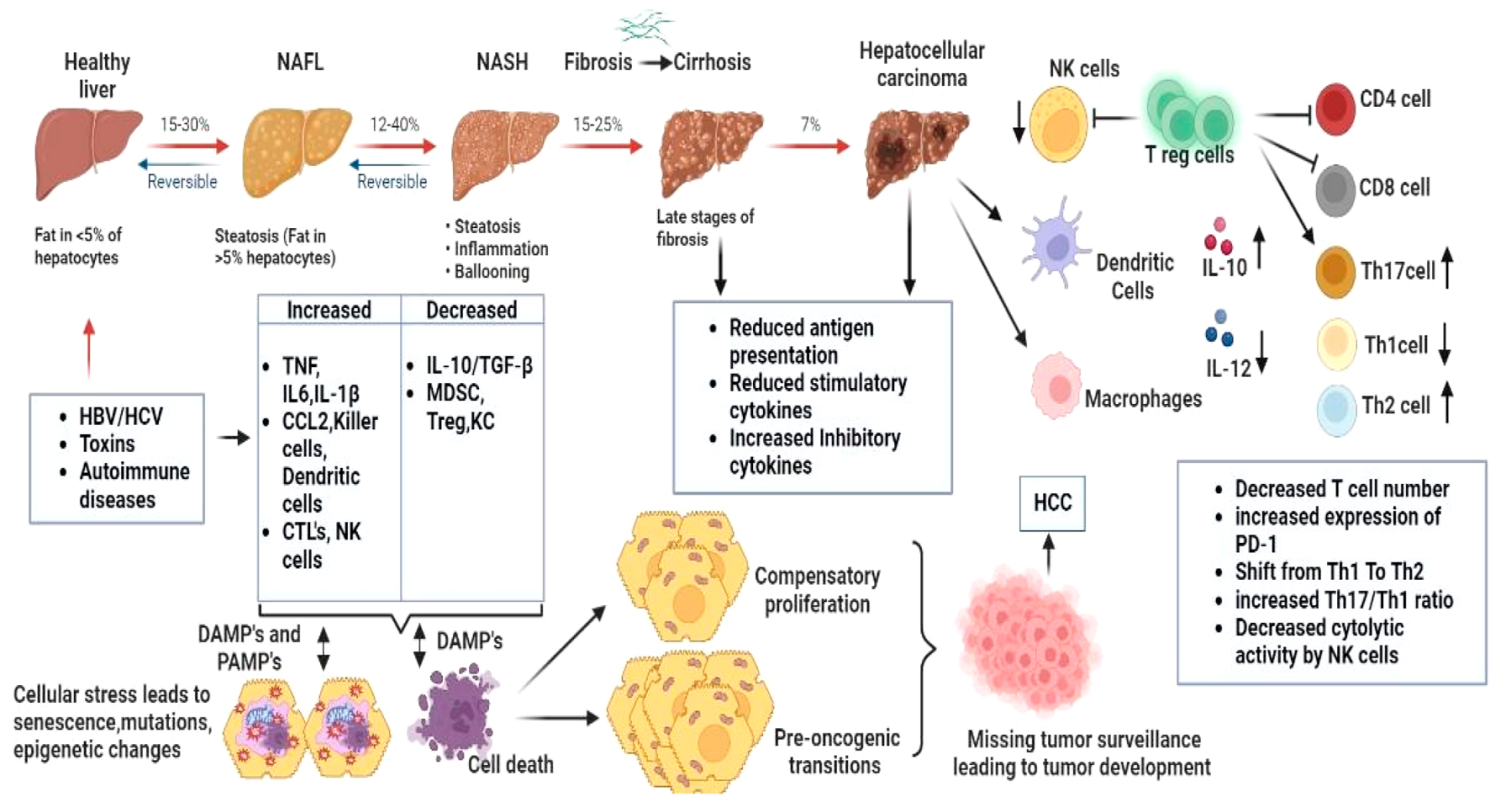
Immunological pathways associated with different risk factors in HCC progression, which are crucial for the development of targeted therapies and interventions to prevent or treat liver cancer.

In summary, it can be asserted that immune cells possess the potential to restrict the progression of HCC by regulating the development of NAFLD or impeding the transformation to NAFLD-HCC as depicted in Figure 3. In order to comprehensively discern the actions of distinct immune cell types, it is imperative to formulate strategies for the temporal modulation of specific immune cell populations, as summarized in Table 2, which represents the roles of different immunological cells in HCC.

**Table 2:** Roles of Immune Cells and Their Clinical Relevance in the Tumor Microenvironment and HCC Disease Progression

Cell Type	Function in Tumor Microenvironment and References	Clinical Role and Significance	Nature of Role
Hepatic stellate cells (HSCs)	Decrease lymphocyte infiltration in tumor^167^	Promote hepatic fibrosis and HCC relapse^168,169^	Promotes tumor growth
	Direct monocytes toward immunosuppression^170^	Reduce immune surveillance and clearance of pathogens	
	Promote tumor angiogenesis	Promote tumor metabolism	
Endothelial cells	Involved in angiogenesis as they express pro-angiogenic receptors^171^	Exhibit increased mortality	
	Exhibit rapid cell division	Enhanced tumor growth	
Cancer-associated fibroblasts (CAFs)	Growth factor production example-EGF^172^	Enhanced tumor cell proliferation	
	Tumor encapsulation^174^	Increased mortality^173^	
	Promotes hepatocarcinogenesis	Increased collagen production^171^	
Myeloid-Derived suppressor cells (MDSCs)	Lead to suppression of non-regulatory T cell response^175^	Marks of advanced hepatic cancer^175^	
Tumor-associated macrophage (TAM)	Releases growth factors and cytokines	Increases tumor size^170^	
	Recruits Treg cells and leads to immunosuppression^177^	Decreases survival rate^176^	
	Promotes M2 macrophage		
	Controls M1 macrophage		

Kupffer cells (KCs)	Foreign pathogen clearance via phagocytosis	Perform tumor surveillance	*Anti-tumor Roles*
			• Role in innate immune response.
Natural killer (NK)	Causes direct tumor cytotoxicity^178^	Reduces tumor growth^179^	• Acts as first line of defense
Dendritic cells (DCs)	Act as antigen presenting cells (APCs)	Exhibit tumor clearance role^180^	• Limited specificity
	Plasmacytoid D has role in immune tolerance		• No memory
	Conventional DC has role in immune suppression^180^		

T cells	CD4/Th1 promotes inflammation^181^	Cause immune cell infiltration leading to rubor, tumor, calor, dolor	*Anti-tumor/Tumor-Promoting Factor Role*
	CD4/Th2 promotes tumor growth	Generate higher mitochondrially derived reactive oxygen species (ROS) levels, cause more oxidative damage^158^	• Role in adaptive immune response
	CD8 Tc, Tumor cytotoxicity	Causes tumor cell death	• Pathogenic specificity
	Treg cells, overall immune suppression^182^	Bad prognosis post-resection	• Memory is generated
B cells	Antibody production	Major role is production of antibodies and memory cells^181^	
	Breg promotes immune suppression^162^	Accelerate tumor progression	
	CD5 promotes tumor growth^183^	Binds to IL-6 and induces a feed-forward loop with STAT3 in B cells to promote cancer	
	CD20 causes direct tumor toxicity^184^	Inhibit tumor cell growth	

## Conclusion and Future Perspectives

HCC is a prevalent form of malignancy that presents a significant global health burden. The escalating prevalence of this condition denotes a significant health concern that warrants attention. This Review provides a comprehensive overview of our current understanding of HCC. We have discussed the major risk factors, the crosstalk between immune cells and tumor cells, available diagnostic approaches, and treatment options. The complexity of HCC has been made evident with the multifactorial interactions involving environmental, genetic, and viral factors, particularly HBV and HCV. It is recommended that individuals who are potentially vulnerable to preventable environmental constituents, including alcohol exploitation and exposure to aflatoxin, be educated on the necessary measures to reduce associated risks. Diagnostic advancements involving imaging techniques and molecular biomarkers have provided improved early detection and prognosis, which are key for the treatment of HCC. Some of the particular diagnostic developments and biomarkers that have been essential in early detection include the following:

**Imaging methods**

- Multiphase computed tomography (CT) and magnetic resonance imaging (MRI) may characterize tiny lesions based on contrast enhancement patterns and produce detailed liver images.
- Contrast-enhancing ultrasound can be used to identify and classify liver lesions based on their vascularity and contrast uptake.^185^
- DCE-MRI, or dynamic contrast-enhanced MRI, provides a real-time blood flow assessment that enables the detection of early-stage malignancies based on their perfusion characteristics.

**Serum biomarkers**

- Alpha-fetoprotein (AFP)
- Des-gamma-carboxy prothrombin (DCP)
- Alpha-fetoprotein (AFP)-L3
- Glypican-3 (GPC3)^186^

**Liquid biopsies**

- Analyzing circulating tumor DNA (ctDNA), RNA, or proteins in the circulation^187^

**Fibrosis evaluation**

- Advanced methods including elastography (e.g., FibroScan) and serum markers (e.g., FibroTest, APRI) can help pinpoint people with cirrhosis or advanced liver fibrosis who are more likely to develop HCC.^188^

**DNA molecular profiling**

- Modern genomic sequencing tools have made it possible to identify particular genetic abnormalities linked to HCC, offering prospective therapeutic targets and assisting in diagnosis.^189^

Other promising therapeutic strategies in HCC management are surgical resection, liver transplantation, and targeted and locoregional therapies.

The management of different types of cancer has shown significant improvement through administering checkpoint inhibitors, particularly anti-PD-1^190^ and anti-CTLA-4^191^ antibodies. Despite being widely accepted, this strategy is hindered by the inadequate presence of tumor-specific antigens (TSAs) to advance highly potent chimeric antigen receptors. Alternative approaches to adaptive cell therapy, such as cytokine-induced killer cells,^192^ tumor-infiltrating lymphocytes,^193^ and NK cells,^194^ have been overlooked due to their inherent non-specificity and complex isolation process.

The efficacy of tumor vaccines in HCC treatment has been hampered by the immune tolerance exhibited by tumors and the limited quantity of TSAs. Immune tolerance reduces the body’s ability to identify cancer cells as foreign substances, which reduces the effectiveness of tumor vaccinations. Finding enough TSAs that are tailored to the tumor is necessary to overcome this difficulty. Although the lack of TSAs can be a barrier, continuing research is concentrating on methods to boost tumor vaccines’ immunogenicity and increase their efficiency in the therapy of cancer. However, DC vaccines hold considerable promise in this regard, owing to their robust ability to present antigens.^195^

The investigation and analysis related to oncolytic viruses are limited.^196^ The paramount concern in the administration of viruses is safety, followed by efficacy. Oncolytic viruses present a considerable challenge to find an optimal balance between safety and toxicity.

Personalized approaches tailored to the specific characteristics of each patient hold great promise for optimizing HCC treatment outcomes. For instance, genetic testing can help in selecting targeted medications, and taking into account liver function can help in deciding whether severe treatments are necessary. These individualized strategies maximize therapeutic efficacy while minimizing side effects, ensuring that each patient receives the most appropriate and efficient care for their particular disease. The incorporation of genomic profiling, molecular subtyping, and emerging immunotherapeutic strategies may revolutionize HCC disease management and overall survival rates. However, challenges remain in fully combating this aggressive disease because of its complex nature and recurrence. Improved understanding of key points like tumor heterogeneity, tumor microenvironment and its interaction with host immune cells, immune invasion of tumor cells, and proliferation strategies will pave the way for novel therapeutic interventions. Hence, further research is needed to unravel the intricate molecular mechanism underlying the development and progression of HCC, which would lead to better prognosis and early detection of the disease, leading to overall decreased mortality rates. Additionally, efforts should be made to direct effective screening programs and spread awareness to facilitate early diagnosis and intervention.

## Abbreviations

HCC: hepatocellular carcinoma
ALD: alcoholic liver disease
NASH: non-alcoholic steatohepatitis
HBV: hepatitis B virus
HCV: hepatitis C virus
DC: dendritic cell
TSA: tumor-specific antigen
COX2: cyclooxygenase-2
NK: natural killer
PD-1: programmed cell death protein-1
NET: neutrophil extracellular trap
CTLA-4: cytotoxic T-lymphocyte-associated antigen 4
ROS: reactive oxygen species
APC: antigen-presenting cell
KC: Kupffer cell
IR: insulin resistance
T2DM: type 2 diabetes mellitus
AFB1: aflatoxin B1
AFP: α-fetoprotein
STAT3: signal transducer and activator of transcription 3
HFD: high-fat diet
